# Nonparametric Frequency Response Identification for Dc-Dc Converters Based on Spectral Analysis with Automatic Determination of the Perturbation Amplitude

**DOI:** 10.3390/s21093234

**Published:** 2021-05-07

**Authors:** Marlon Granda, Cristina Fernandez, Andres Barrado, Pablo Zumel

**Affiliations:** Power Electronics Systems Group (GSEP), Department of Electronic Technology, Universidad Carlos III de Madrid, 28911 Leganes, Spain; mgranda@ing.uc3m.es (M.G.); cfernand@ing.uc3m.es (C.F.); barrado@ing.uc3m.es (A.B.)

**Keywords:** digital control, power converters, identification

## Abstract

Digital control for high switching frequency converter enables new features on DC-DC power conversion for a minimum cost. Frequency response identification is one such enabled functionality used in auto tunning, measurement of components to assess the converter’s state of health, or system stability monitoring. High accuracy, flexibility to operate in open or closed loop, and minimum impact in the converter’s regular operation are the frequency response identification system’s goals. We propose in this paper a nonparametric identification system addressing these main goals. First, it can autoadjust the perturbation size to reduce the perturbation’s impact on the converter’s output quantities. Second, as it is based on spectral analysis, it is suitable for open and closed-loop operation. Third, we demonstrate the identification system’s high accuracy, achieving a very low difference between the experimental measurements and the discrete model used as reference.

## 1. Introduction

The control of high-frequency power converters has suffered an evolution in recent years due to the newly available high-end digital devices, with outstanding performance and reduced cost. These devices allow for an implementation not only of a conventional linear regulator to control the voltage or the current but also to incorporate advanced features that can improve the performance of the converters [1,2], like protections, communication [3], integration on large systems, or identification techniques. The use of identification techniques to measure the frequency response of the converter [4] can be applied for different purposes such as checking the stability of the system [5,6], adapting the regulator to different situations [7,8,9] or monitoring the health status of the converter or load [4,9,10,11,12,13].

In general, the dynamic behavior of switched power converters can be considered reasonably linear around a working point: when introducing a sinusoidal at its input, the output signal is also a sinusoidal signal of the same frequency as the input. Other spectral components associated will appear in the spectrum of the output signal. However, suppose the perturbation frequency is sufficiently lower than the switching frequency, and the perturbation amplitude is sufficiently small. In that case, these components can be discarded and not considered in the system’s dynamic behavior [14], so the converter can be analyzed as a Linear Time-Invariant system. Because of the system’s linearity, it is possible to use broadband signals to shorten the identification process, which excites the system simultaneously at several frequencies in the bandwidth of interest. Such broadband excitation allows time saving during the identification and less impact in the converter operation, compared with single frequency excitation [9]. Some of these signals are the multisine [15,16,17,18,19,20] and the pseudorandom binary sequence (PRBS) [5,6,8,10,21,22,23]. This work is focused on this last option because it can be easily generated with a shift register and a logic gate, so it is very suitable for its implementation on a digital device.

The implementation of identification techniques can be done using digital control platforms such as FPGAs [2,5,10,21,22,24], microcontrollers [9,25] or Systems on Chip (SoC) [26,27,28]. FPGA implementation allows for task parallelization and a very accurate timing. Microcontrollers are a low cost alternative, and some algorithms can be easier to implement by programming than by hardware description in an FPGA. However, they can be limited in very accurate timing control and task parallelization, depending on the device peripherals. Since SoC implementation can benefit from programming in the microprocessor cores and from paralleling and timing control in the programmable logic system, this technology has been selected to implement the identification system proposed on this work.

Regarding the design of the perturbation signal, critical parameters in the design of the PRBS are its length (related to the number of bits), its amplitude and the frequency at which it is generated (clock frequency of the shift register) [29,30]. The length (number of bits N) and the frequency of the shift register ($f_{PRBS}$) determine the minimum perturbed frequency ($f_{min}$) and the maximum frequency ($f_{max}$) in which the PRBS has a flat spectrum [23,24], which can be calculated using (1) and (2). Moreover, all the perturbed frequencies are integer multiple of $f_{min}$. However, it is important to highlight that the perturbation is not a limited bandwidth signal, and there are harmonic components for frequencies greater than $f_{max}$, although their amplitudes decrease with increasing frequency. The amplitude of the PRBS is critical, since it must be low enough to avoid entering nonlinearities and large enough to obtain a good signal-to-noise ratio (SNR) [5]. This amplitude is usually determined by a trial and error process. The identified frequency response contains the model between the input and the output and the effect of noise on the output signal. Due to this noise, together with the identification technique’s implementation, the resulting Bode plot is typically noisy at high frequency, which may be problematic for some applications. For instance, to calculate a compensator, it is critical to know the frequency where the system’s frequency response is 0 dB, which is very difficult with a noisy Bode plot.

(1) $$f_{min}=\frac{f_{PRBS}}{2^{N}-1}$$

(2) $$f_{max}=\frac{f_{PRBS}}{2}$$

Therefore, different authors propose some techniques to smooth the Bode plot:

- Modifying the spectrum amplitude of the perturbation signal by using a pre-emphasis filter [4,31] or performing separate tests with colored noise signals [13]. This way, the frequency components where the system exhibits high attenuation are amplified. For example, in many power converters it is useful to amplify the perturbation high frequency content, because the system behaves as a low pass filter. However, this solution requires specific knowledge of the system’s dynamic response and the implementation of two filters (pre-emphasis and de-emphasis) [4,5]. In this work we do not apply additional filters or colored signals but propose a novel procedure to automatically determine a suitable PRBS amplitude.
- Truncating the impulse response [5]. Cross-correlation methods are usually applied with PRBS excitation, and the result of this nonparametric identification technique is the impulse response. A smoother Bode plot is obtained by truncating such impulse response [5] to eliminate the noisy samples that will produce an irregular frequency response plot. The main disadvantage of this solution is that it requires a perturbation signal long enough to ensure that the truncation only impacts the Bode plot’s noise and does not eliminate part of the system’s dynamic response. Therefore, some knowledge of the system under test is convenient. In this work we use the impulse response as a tool to calculate the most suitable amplitude of the PRBS. The system identification is done using spectral analysis (Fourier analysis of the input and output signals) without requiring any knowledge of the system under test.
- Applying a smoothing filter to the identified Bode plot [13,32]. This easy postprocessing solution is particularly effective for identifying systems that behave as low-pass filters since the noise is usually at high frequency, where there are more frequency components due to the logarithmic representation. Of course, it is essential to ensure that the smoothing filter does not affect the identified system’s dynamic. In this work, because of the accurate synchronization of all the processes in the SoC digital platform, as well as the automatic calculation of the PRBS amplitude, the identification results are good even skipping the smoothing filter. Thus, in our proposal this step is optional.

This work presents a novel nonparametric identification technique to measure a switched DC/DC converter’s frequency response, implemented on a System on Chip (SoC). Regarding previous works [4,5,8,9,10,13], the main contribution of this work is to find a unique combination of different techniques to achieve a noticeable accuracy in the measurement. First, PRBS cross-correlation with impulse response calculation is only used for the automatic determination of a suitable perturbation amplitude. Second, spectral analysis is applied for the identification itself, allowing open and closed-loop operation. Finally, the implementation on a SoC [26,27,28] allows full online operation, integrating all processing task in the digital platform and very accurate timing control.

The main contribution of this work is that, thanks to the automatic calculation of a suitable perturbation and the implementation on a SoC, the resulting Bode plot is smooth and accurate, and other postprocessing mechanisms to reduce the measurement’s noise, which require previous knowledge of the system, can be avoided.

## 2. Proposed Identification Methodology

**Figure 1:**
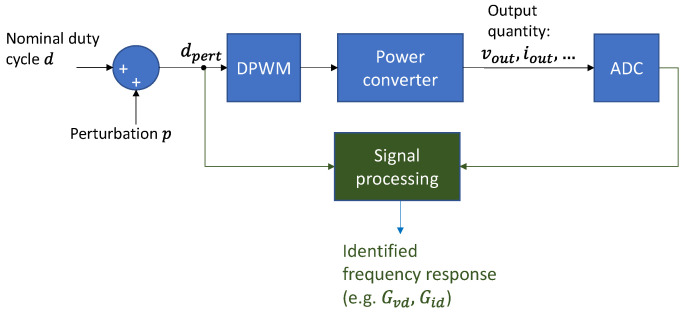
Basic blocks involved in the identification of a power converter.

Figure 1 represents the basic blocks to identify a power converter’s frequency response working open-loop besides the power stage, the digital pulse width modulator (DPWM) and the analogue to digital converter (ADC).

One way to obtain the frequency response of a power converter is by using a broad-spectrum signal as a perturbation signal to later apply the Fourier analysis to the input and output signals to obtain the final Bode. In Figure 1, the control to output transfer function can be calculated as:

(3) $$G_{vd}=FFT\left(v_{out}\right)-FFT\left(d_{pert}\right),$$

where $v_{out}$ is the sampled output voltage, $d_{pert}$ is the sampled input duty cycle, and FFT denotes the Fast Fourier Transform algorithm.

Cross-correlation is used as a nonparametric identification technique to obtain the impulse response of a linear system [5,8,10,22]. One of the characteristics of PRBS is its approximation to white noise, which means that its autocorrelation is an ideal delta function. In Figure 1, assuming that the perturbation p is a PRBS, the cross-correlation $G_{dv}$ between the output voltage and the duty cycle is:

(4) $$G_{dv}\left[n\right]=\overset{N-1}{\underset{k=-N}{\sum }}d_{pert}\prime [k]\cdot v_{out}\left[k+n\right]=h\left[n\right]+G_{dr}\left[n\right]=h\left[n\right],$$

where $v_{out}$ is the sampled output voltage, $d_{pert}\prime$ is the perturbed duty cycle without its DC component (nominal duty cycle), h[n] is the impulse response of the power converter, and $G_{dr}\left[n\right]$ is the cross-correlation between the noise component of the output voltage and the duty cycle. $G_{dr}\left[n\right]$ results in 0 because of the quasi randomness of the PRBS.

In real cases, the system’s frequency response is obtained, and it is also accompanied by the contribution to the output by noise: electrical noise coupled from internal or external sources and quantization noise due to the analogue to digital conversion process. Thus, it is common to use various techniques to minimize noise’s effect on the identification results.

### 2.1. Determination of the Amplitude of the Perturbation Signal

In the proposed system, the PRBS is generated in the digital device. The identification system also incorporates a method to autonomously establish the amplitude of the perturbation for each converter to be identified, as a trade-off to ensure a small-signal test while minimizing the impact of the noise in the measurement. Such a method consists of an iterative process of three steps described in the following subsections.

#### 2.1.1. Identification of the Impulse Response to Characterize the Impact of the Noise

**Figure 2:**
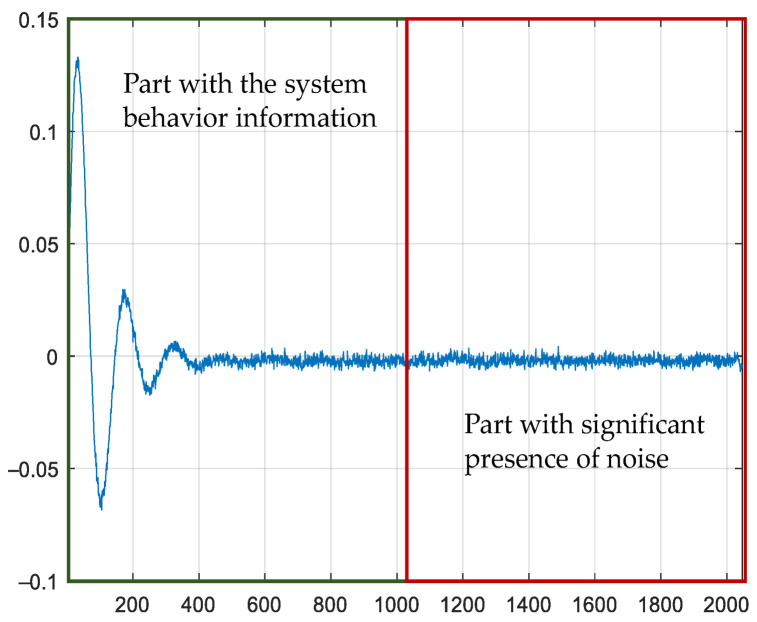
Impulse response with two different areas of information.

The proposed approach requires identifying the system’s impulse response to assess the impact of the noise on the measurement. Cross-correlation is the most suitable technique for identifying impulse response, especially considering that the perturbation signal is a PRBS, whose autocorrelation is a Dirac pulse, as previously explained. The impulse response has two differentiable zones that depend on the dynamics of the converter, as shown in Figure 2:

- The first area is the one that contains the information about the system dynamics.
- The second zone is the one that would ideally be close to zero, but this does not happen due to the presence of noise. The length of this zone depends on the length of the PRBS. It is essential to ensure that the PRBS has enough bits to identify the system’s dynamics and a small zone with no dynamics.

In this work, we will use this second part of the impulse response to assess noise’s influence on the identification result. Note that the impulse response does not necessarily correspond to the transfer function to be identified, as it is only a tool to assess the noise.

#### 2.1.2. Quantification of the Impact of the Noise

The figure of merit to quantify the noise’s impact is the standard deviation σ since it assesses how far a set of data is from its mean value. It can be calculated using (5), where $x_{i}$ are the samples corresponding to the second part of the impulse response, n is the number of samples of this vector, and $\bar{x}$ is its mean value.

(5) $$\sigma =\sqrt{\frac{1}{n-1}\overset{n}{\underset{i=1}{\sum }}{\left(x_{i}-\bar{x}\right)}^{2}}$$

It is not possible to determine what part of the impulse response includes the system’s dynamics and what part has negligible information about the system since it depends on the dynamics of the system to be identified. In this work, we calculate the standard deviation of the second half of the impulse response. The reason is that, although the second area may contain some information from the converter’s dynamic, the amplitude of the noise has a higher impact than in the first part. The mean value $\bar{x}$ is zero in the calculation of this standard deviation.

#### 2.1.3. Analysis of the Standard Deviation of the Second Part of the Impulse Response

**Figure 3:**
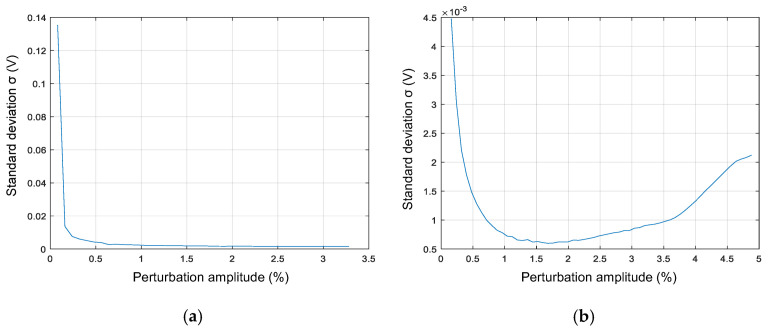
Example of the evolution of the standard deviation of the noise (second part of the impulse response): (**a**) buck converter; (**b**) boost converter.

The analysis of σ evolution is an iterative process. The identification system starts the procedure with the minimum considered amplitude. For each amplitude of the perturbation, the impulse response is obtained, and then the standard deviation of the second part of the impulse response is calculated. The main idea is to evaluate the evolution of the standard deviation in every iteration. In the first iterations, σ is expected to decrease significantly as the perturbation signal’s amplitude is increased, as shown in Figure 3. Nevertheless, after the first iterations and this negative slope, two cases can generally occur:

- The reduction of σ is minimum in the last iterations, and therefore, increasing the amplitude of the perturbation signal has a negligible impact on improving the identification result. This is the expected case in converters with a reasonably linear dynamic behavior, such as the buck converter, Figure 3a.
- The standard deviation increases with the amplitude of the perturbation. This is expected in converters with a dynamic behavior dependent on the working point, such as the boost converter, reaching the results shown in Figure 3b.

The iteration ends when the standard deviation meets different criteria: there is a positive trend or the improvement compared to the previous step is lower than a given threshold. The lower the value of parameter σ, the better the identification results but also the longer the time to complete the iterative process.

### 2.2. System Identification Using Spectral Analysis

The cross-correlation technique is very appropriate to identify a system when the input signal of the system is directly the PRBS perturbation. This is the case of the identification of the control to output signal transfer function [5]. The problem arises when the perturbation is produced in the system, but it is not the input signal of the system under test. In these cases, if using the cross-correlation technique, other additional processes will be required.

**Figure 4:**
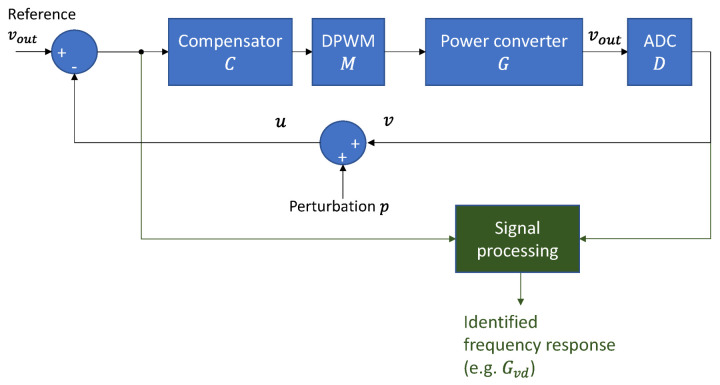
Block diagram of a power converter working closed-loop and injecting a perturbation p.

Figure 4 shows the basic blocks to identify a power converter working in closed-loop control. In this case, the cross-correlation of the input of the system u and its output v does not directly yield the impulse response of the open-loop gain. The reason is that the input signal u does not have an autocorrelation equal to the Dirac impulse because it is not a PRBS like the perturbation p, as shown in Equations (6) and (7). On the other hand, if spectral techniques are considered, i.e., the direct application of the Fourier Transform to the input signal u and the output signal v, the frequency response is obtained directly, as can be deduced from Equations (7)–(9).

(6) $$G_{OLG}=C\cdot M\cdot G\cdot D$$

(7) $$u=\frac{1}{1+G_{OLG}}\cdot p$$

(8) $$v=-\frac{G_{OLG}}{1+G_{OLG}}\cdot p$$

(9) $$G=\frac{v}{u}=-G_{OLG}$$

In the proposed identification system, the cross-correlation technique has been replaced by spectral analysis, performing the Fourier analysis of the signals involved in the identification process. Such an approach allows the system to identify every block’s Bode plot, even though its input is not a PRBS.

### 2.3. Postprocessing of the Measure: Smoothing Process

Due to the noise present at high frequency in the identification frequency response, a smoothing process can be optionally applied to the module and phase obtained, thus achieving a smoother result without sudden variations in the identified frequency characteristics. This process is optional because, as will be seen in the experimental results, the Bode plot obtained in many cases is sufficiently clean. The smoothing process is done by applying the moving median.

Since the identification result is represented on a logarithmic scale, the density of points increases as the frequency increases. Therefore, the application of smoothing cannot be carried out proportionally over the entire frequency range since an alteration of the Bode plot would be produced. For this reason, a system of segments is used. The number of points included in each segment is determined by (10). Smoothing is applied to each segment separately, considering a different window length to apply the median. The length of the window is set by (11), where: $L_{vector}$ is the length of the data vector to which the smoothing process will be applied; $N_{sg}$ is the number of segments into which the data is divided; i is the segment number for which the number of points and window length are calculated, and $w_{o}$ is the starting window size of the moving median.

(10) $$Sg_{i}=\{\begin{array}{c} \frac{L_{vector}}{2^{N_{sg}-i}}-Sg_{\left(i-1\right)},\;\;\;\;\;\;\;\;\;\;i<3\\ \frac{L_{vector}}{2^{N_{sg}-i}}-Sg_{\left(i-1\right)}\cdot 2,\;\;\;i\geq 3 \end{array}\;\;\;,$$

(11) $$w_{i}=w_{o}{}^{i-1},$$

**Figure 5:**
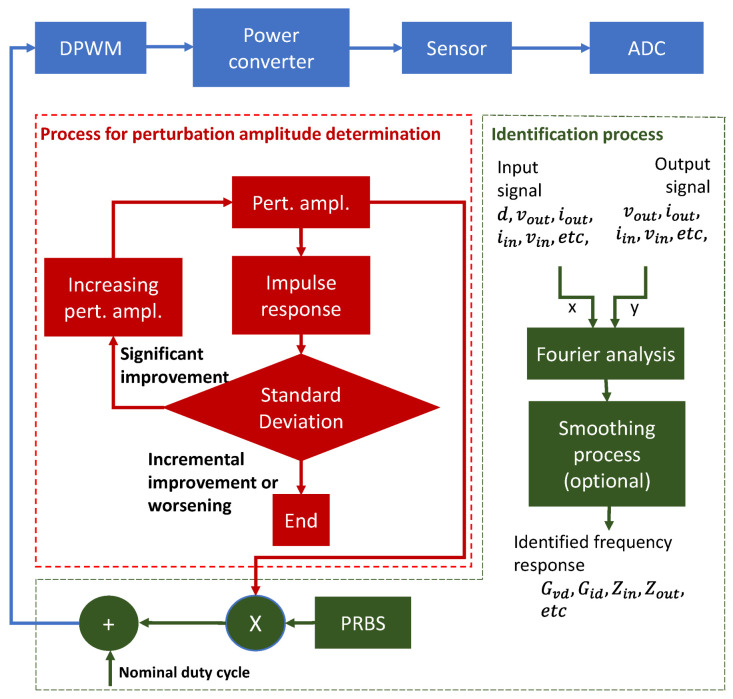
Complete block diagram of the proposed identification system for open-loop identification.

**Figure 6:**
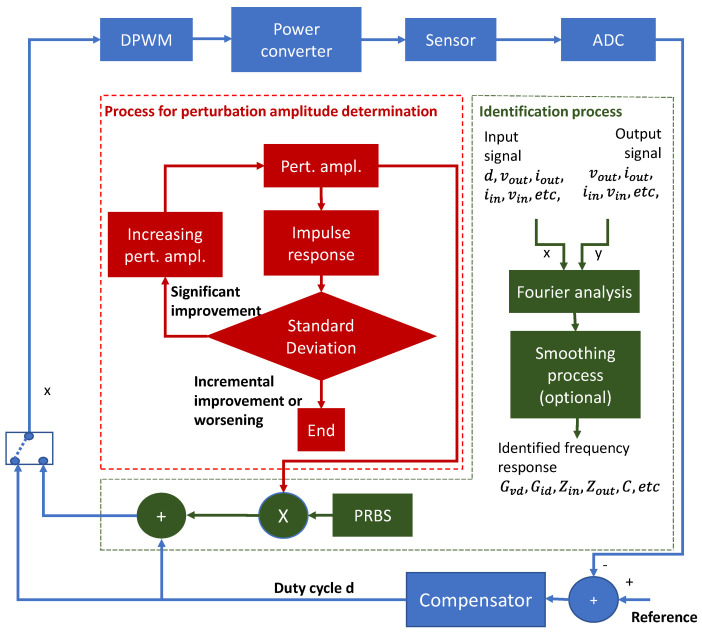
Complete block diagram of the proposed identification system for closed-loop identification.

The identification systems containing all described blocks are shown in Figure 5 and Figure 6 for open and closed-loop. All blocks, other than the power converter, sensor, and ADC, are implemented in an SoC.

The most important red and green blocks in Figure 5 and Figure 6 perform the following processes:

- Fourier analysis: this block applies the FFT to its input signals and calculates the difference among the transformed signals to obtain the frequency response. Expression (3) is a particular case of this block when the input signals are the perturbed duty cycle and the output voltage.
- Smoothing process: it consists of the application of moving median and expressions (10) and (11) to the frequency response obtained in the block “Fourier analysis”.
- The block impulse response applies expression (4) to the PRBS and another signal. In this particular work we have chosen the output voltage to be cross-correlated with the PRBS.
- The standard deviation analysis block applies expression (5) to the impulse response calculated in the previous block.

### 2.4. Simulation of the Identification Procedure

**Figure 7:**
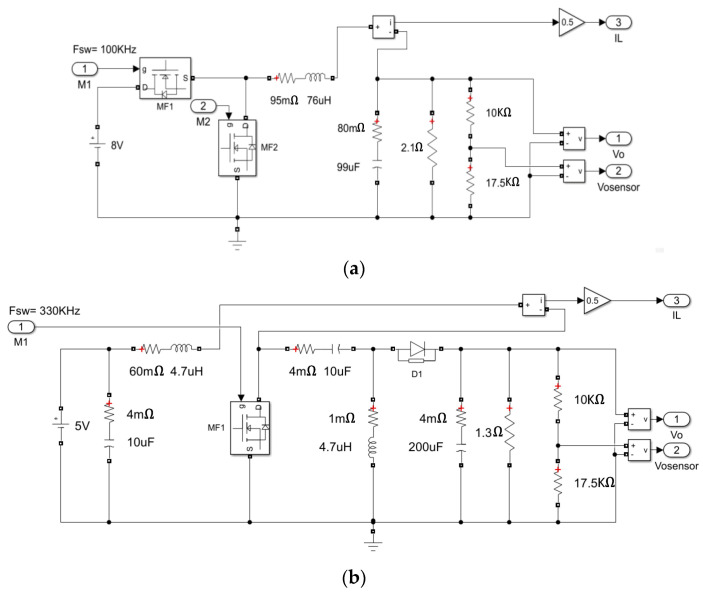
Matlab-Simulink schematics of the simulated converters with the corresponding parameter value. (**a**) Buck converter. (**b**) SEPIC converter.

**Figure 8:**
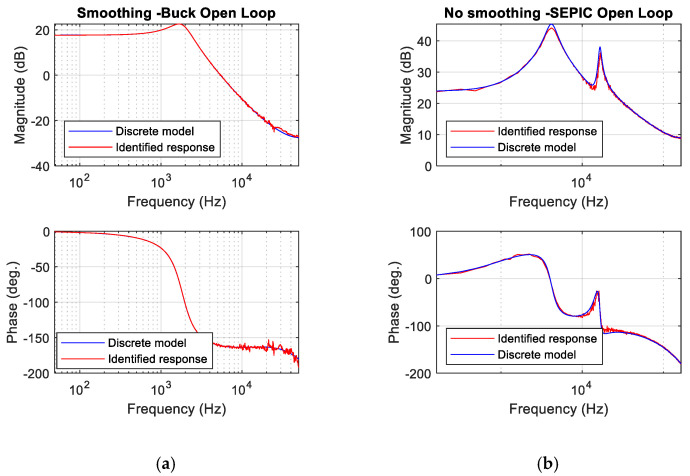
Simulation of the proposed identification procedure. (**a**) Control to output voltage transfer function for the buck converter in Figure 7a. (**b**) Control to inductor current transfer function for the SEPIC converter in Figure 7b.

The proposed identification method has been validated through simulation with Matlab Simulink. Two different topologies have been simulated (Figure 7), producing the PRBS signal (11 bit, register clock at switching frequency) and performing a Fourier analysis of the controlled signal and the perturbation signal to calculate the Bode plot. The simulation results have been compared to the discrete theoretical model of the converter [14], computed also using Matlab (Figure 8).

In the first case, the identified plant is the control to output voltage transfer function of a buck converter. This is a second-order transfer function, and the simulation results match very well the discrete model.

In the second case, the tested converter is a SEPIC [33]. The identified system is the control to inductor current transfer function, which is a fourth-order transfer function. The identified Bode plot matches very well the results of the discrete model as well (Figure 8).

These simulations show the feasibility of using the PRBS and the spectral analysis to identify the dynamic response of this kind of DC/DC converters.

## 3. Results

**Figure 9:**
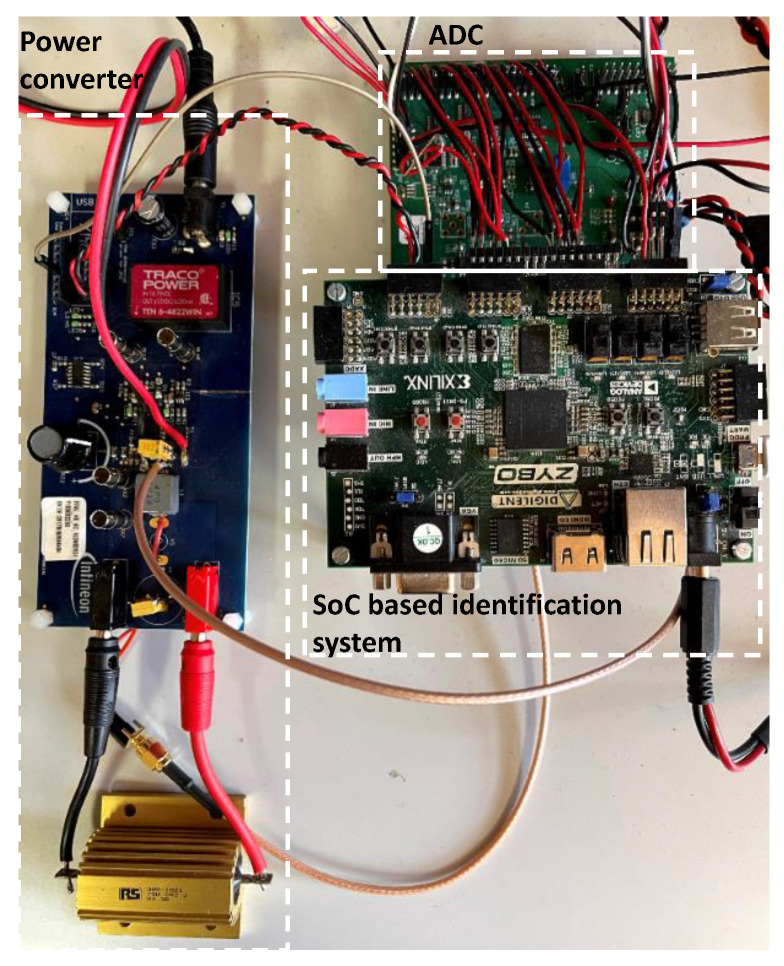
Setup for identification. Three different power converters have been tested, while the digital control card based on an SoC is the same for all tests.

This section presents the results obtained using the identification system described in the previous sections. A variety of buck and boost type converters (Table 1) have been employed to perform the experimental tests and validate the system’s operation. The picture in Figure 9 corresponds to the experimental setup for one of the converters.

As highlighted as one of the novelties of this work, the identification system and the digital control have been implemented on a System on Chip (ZYNQ-7010 in a Zybo board in Figure 9), including in the same digital device a microprocessor and an FPGA communicated by an internal bus.

Signal processing algorithms can be easily developed and debugged in a microprocessor. Thus, the perturbation signal generation, the identification processes (spectral analysis), and the perturbation size are implemented in the SoC microprocessor. Other algorithms related to the autodesign of compensator [2] or estimating the value of the converter components for health assessment [34] can be easily included in the digital control device. Critical tasks with time constraints can be implemented in the FPGA, warranting a tight control of timing. In this case, the DPWM, ADC interface, and the compensator are implemented with specific hardware into the FPGA. Dead times or multiphase modulators, hardware filters for analogue to digital conversion, and hardware protections could also be included.

The digital control system is autonomous as all calculations are performed in the SoC. An HMI has been designed in Matlab to interface the digital control, retrieve experimental data, and debug the system. Consequently, the SoC provides remarkable flexibility for the implementation of the proposed identification system.

### 3.1. Validation of the Implemented System to Automatically Determine the Amplitude of the Perturbation Signal

**Figure 10:**
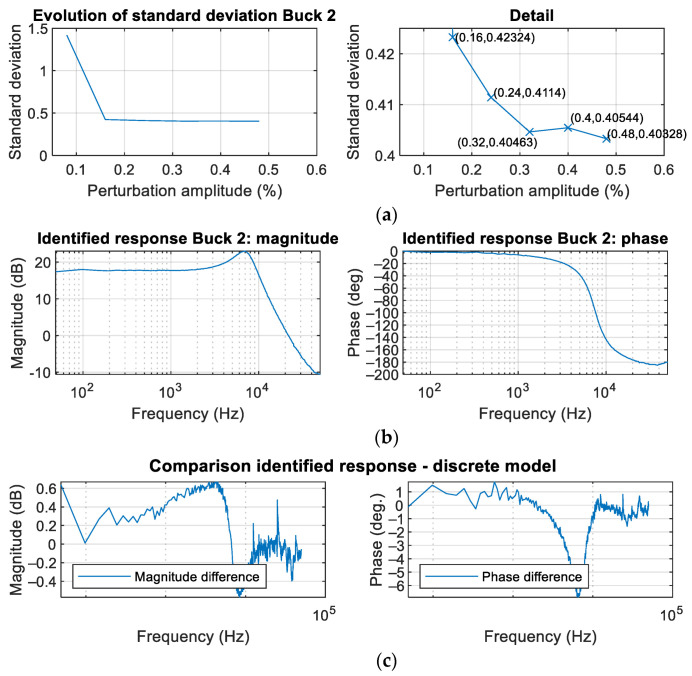
Determination of the perturbation amplitude for converter Buck 2 (Table 1). (**a**) Evolution of the standard deviation. (**b**) Identified frequency response. (**c**) Difference between the identified frequency response and discrete theoretical model.

Figure 10 illustrates the mechanism to determine the amplitude of the perturbation signal. Figure 10a shows the evolution of the standard deviation (1) as a function of the perturbation amplitude for the converter Buck 2 (Table 1). The minimum value of the amplitude perturbation corresponds to a single count of the DPWM, i.e., 1/1250 = 0.08%, as the maximum value of the DPWM counter is 1250. The first iteration provides a significant reduction of the standard deviation: from 1.418 to 0.42324. The additional plot in Figure 10a shows a vertical zoom. The system increases the perturbation size by 0.08% in each iteration. The stop criterion is to find two consecutive iterations with an increasing value of standard deviation or to find a reduction lower than 2% compared to the previous iteration. This last condition is the case of this example, and the identification results applying the determined perturbation amplitude (0.48%) are shown in Figure 10b. The indicators to assess the identification quality are the differences between identified frequency response and the converter’s discrete model [14] both in magnitude and phase. Figure 10c shows a difference in magnitude within ±0.5 dB and in phase within (−6.5 deg.,+1 deg.), which can be considered reasonably accurate. Moreover, the measured Bode plot is very smooth so that the optional smoothing filter can be skipped.

### 3.2. Validation of the Implemented Identification System

**Figure 11:**
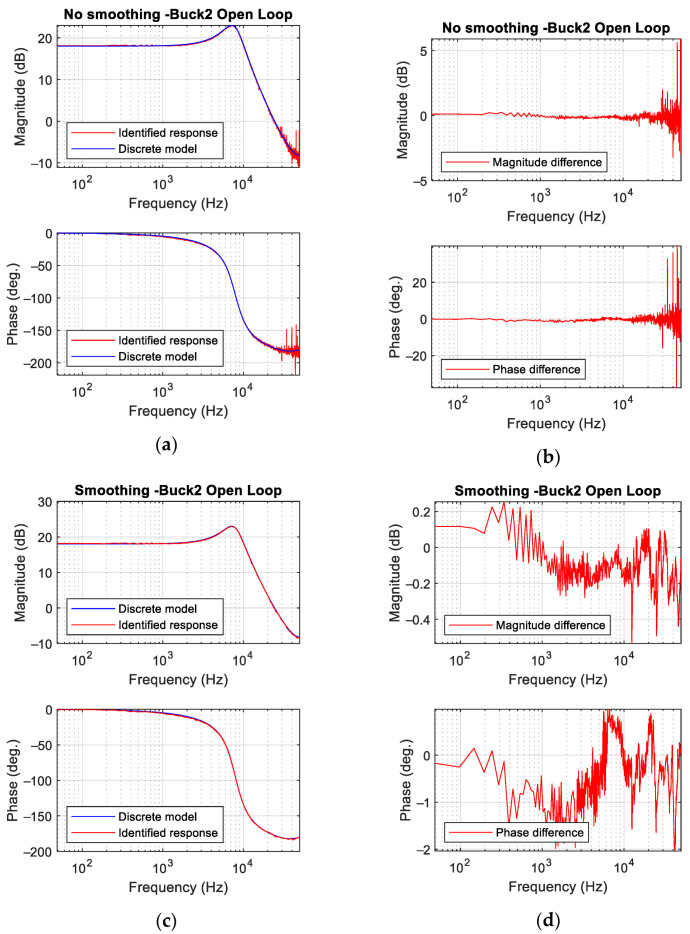
Buck converter open-loop results. (**a**) Bode plot without smoothing process. (**b**) Difference between discrete theoretical model and experimental measurements without smoothing process. (**c**) Bode plot with smoothing process. (**d**) Difference between model and measurement with smoothing process.

Figure 11 shows the identification results of converter Buck 2 (Table 1) for open-loop operation. In this case, the size of the perturbation is 3.2% in terms of the duty cycle. Figure 11a corresponds to the identified Bode plot without using the smoothing process. The experimental measurement overlaps the discrete model [14] of the buck converter. Matching between the theoretical model and experimental results is good, and the main differences appear at high frequency (30 kHz approximately). Figure 11b plots the difference between the theoretical model and the experimental results, and it confirms a good matching up to 30 kHz. Compared with other results shown in the literature [1,5,11,12], the noise in the frequency response is considerably low. Notice that the results in Figure 11a,b skip the smoothing process.

Figure 11c,d show results from the same test but using the smoothing process described in Section 2. The matching between the experimental Bode plot and the theoretical model is remarkable (Figure 11c): the difference in magnitude is within ±0.25 dB and in phase within ±2 deg. (Figure 11d).

**Figure 12:**
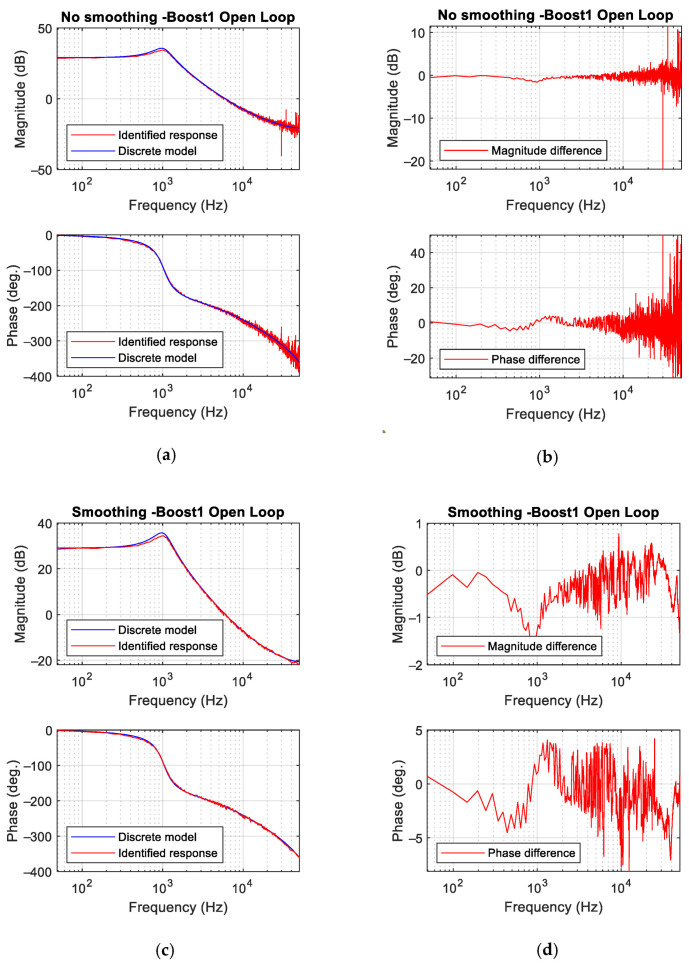
Boost converter open-loop results. (**a**) Bode plot without smoothing process. (**b**) Difference between discrete theoretical model and experimental measurements without smoothing process. (**c**) Bode plot with smoothing process. (**d**) Difference between model and measurement with the smoothing process.

Figure 12 shows the identification results of the converter Boost 1 (Table 1), for the same perturbation than in the previous case. As shown for the buck converter, the smoothing process is only applied in Figure 12c,d. Without the smoothing process, results are reasonably good up to 20 kHz approximately. When the smoothing process is applied, matching between the theoretical and the identified frequency response is remarkable: magnitude difference is within ±1.5 dB and in phase within ±7 deg. Even if these results are not so accurate as in the buck converter, they can be considered very good.

**Figure 13:**
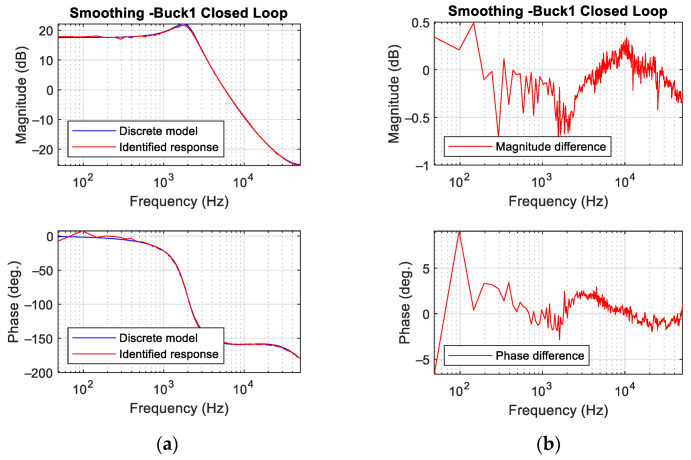
Results for Buck1 converter working closed loop. (**a**) Bode plot with smoothing process. (**b**) Difference between theoretical discrete model and experimental measurements with smoothing process.

Finally, the identification results of Buck 1 (Table 1) working closed loop are shown in Figure 13 for the same perturbation than in the previous case. A PID compensator is tuned to achieve a cross-over frequency $f_{c}=3\;\mathrm{kHz}$ and a phase margin PM=50°. The results corresponding to the power stage identification applying the smoothing process are shown. The magnitude difference is within ±0.5 dB and in phase within ±7 deg. These results confirm the remarkable accuracy of the identification even working in closed loop.

## 4. Discussion and Conclusions

In this work, we propose a novel approach for the nonparametric identification of the frequency response of DC/DC converters, based on a digital implementation on an SoC.

The main differences concerning similar works lie in the reduction of the number of processes required in the identification process (truncation, digital filters), an automatic design of the perturbation, and the use of spectral techniques to perform the nonparametric identification. Moreover, the entire proposed system is implemented online on an SoC. The main advantages of using a SoC are the multitask operation, the easy implementation of the processing, and the accurate synchronization of all the processes. This technology allows for the running of the identification process while the control is regularly working. Furthermore, it takes advantage of the high resolution of the DPWM implemented on the programmable logic system of the SoC, with accurate control of the sampling. The result is a precise synchronization of the control, the sampling, and the identification processes.

The system can automatically determine the amplitude of the perturbation through an iterative process. The calculation is based on the obtention of the impulse response using the cross-correlation of the perturbation and the output signals. The impact of the noise is assessed calculating the standard deviation of the amplitude of the second part of this impulse response. The proposed process has been successfully applied to both actual buck and boost converters, despite the significant differences in their dynamic behavior. Whereas other systems in the state of the art may require a manual adjustment of the perturbation amplitude, based on an empirical approach, the presented approach provides an automatic design of a suitable perturbation.

The nonparametric identification of the system is done by applying spectral techniques (Fourier analysis of the input and output signals). This way, the frequency response of the converter can be periodically calculated without a significant impact on the regular operation of the converter, which can be working open or closed loop.

The experimental results show a remarkable accuracy of the identified Bode plot, both in open and closed loop, compared to the discrete theoretical model. The accurate and smooth Bode plot allows this system to be used in different applications: the self-design of regulators ([7]) and also in applications of parametric identification of converter components for the evaluation of the health status of the converter ([34]).

## Figures and Tables

**Table 1:** Prototypes’ data.

Parameter	Buck 1	Buck 2	Boost 1
Inductance (µH)	76	4.85	64.6
Output capacitor (μF)	99	84.73	95
Load resistance (Ω)	10	2.5	14.6
Nominal duty cycle (%)	50	50	50
Input voltage (V)	8	8	8
Switching frequency (kHz)	100	100	100

## Data Availability

Not applicable.
